# Translation and Validation of the Japanese Version of the Dialysis Patient‐Perceived Exercise Benefits and Barriers Scale

**DOI:** 10.1111/jorc.70076

**Published:** 2026-08-03

**Authors:** Ryota Kumakura, Keiko Tasaki, Tomomi Horiguchi, Yuya Asada, Yukari Fujita, Noboru Fujino

**Affiliations:** ^1^ Faculty of Health Sciences, Institute of Medical Pharmaceutical and Health Sciences Kanazawa University Ishikawa Japan

**Keywords:** cross‐cultural comparison, exercise, haemodialysis, health promotion

## Abstract

**Background:**

Regular exercise is recommended for patients undergoing haemodialysis; however, participation rates remain low in Japan. Perceived benefits and barriers are key cognitive determinants of exercise behaviour, yet no validated instrument exists to assess these perceptions among Japanese patients receiving haemodialysis.

**Objectives:**

Translate and culturally adapt the Dialysis Patient‐Perceived Exercise Benefits and Barriers Scale into Japanese and evaluate its psychometric properties.

**Design:**

A methodological study.

**Participants:**

In total, 150 adults undergoing maintenance haemodialysis were recruited from facilities across Japan.

**Measurements:**

Structural validity was examined using exploratory and confirmatory factor analyses. Concurrent validity was assessed through correlations with physical activity levels measured using the International Physical Activity Questionnaire. Known‐groups validity was evaluated by comparing scale scores between participants with and without exercise habits. Reliability was assessed using internal consistency and item–total correlations.

**Results:**

Confirmatory factor analysis supported the original six‐factor structure with acceptable model fit (CFI = 0.922, TLI = 0.910, RMSEA = 0.055 and SRMR = 0.062). The Japanese version showed moderate concurrent validity with physical activity levels (Spearman's *r* = 0.379, *p* < 0.01) and discriminated between participants with and without exercise habits (*p* < 0.001). Internal consistency was excellent (Cronbach's *α* = 0.901), and all item–total correlation coefficients exceeded acceptable thresholds.

**Conclusions:**

The Japanese version of the Dialysis Patient‐Perceived Exercise Benefits and Barriers Scale is a reliable and valid instrument for assessing exercise‐related perceptions among patients receiving haemodialysis in Japan. This tool may facilitate individualised exercise counselling and support culturally appropriate exercise interventions in renal care.

## 1 Introduction

Chronic kidney disease (CKD) is a significant global public health issue, affecting over 9.1% of the global population (Deng et al. 2025). CKD can progress to kidney failure requiring kidney replacement therapy. Currently, an estimated 4.5 million people worldwide are living with kidney failure requiring kidney replacement therapy, a burden projected to exceed 5 million by 2030 (Liyanage et al. 2015). Haemodialysis (HD) remains the most widely used kidney replacement therapy worldwide, although associated challenges vary across countries depending on demographic trends and healthcare infrastructure.

Japan represents a distinctive context, with more than 340,000 individuals undergoing dialysis and one of the highest prevalence rates per million population globally. Patients receiving HD in Japan are typically older, have longer treatment durations and bear a high burden of comorbidities, including diabetes and cardiovascular disease (Hanafusa et al. 2024). Frailty increases with dialysis duration, reaching 36.2% among patients treated for more than 30 years, and is associated with an increased risk of becoming bedridden (Yamamoto et al. 2024), highlighting physical vulnerability as a major clinical challenge in ageing populations undergoing dialysis.

Exercise is now widely recognised as a critical adjunct to HD care. Regular physical activity improves muscular strength and cardiovascular capacity, as well as mental health and quality of life, while reducing fatigue (Huang et al. 2019; Wahida et al. 2023; Salhab et al. 2019). Higher physical activity levels are associated with lower hospitalisation and mortality rates in this population (Martins et al. 2021; Yamamoto et al. 2021). Despite these benefits, exercise participation remains low in Japan, with 60%–80% of individuals receiving dialysis reporting minimal or no regular physical activity (Nitta et al. 2021). This persistent gap between evidence and practice underscores the need to better understand the factors influencing exercise behaviour in this population.

## 2 Literature Review

Behavioural theories offer useful frameworks for understanding low exercise participation among patients undergoing HD. The Health Promotion Model (HPM) posits that health behaviours are shaped by cognitive factors, particularly perceived benefits and barriers (Pender et al. 2014). In this population, perceived barriers often outweigh perceived benefits, reducing motivation to engage in exercise (Lightfoot et al. 2021; Ghafourifard et al. 2021).

Several instruments have been developed to assess exercise‐related perceptions. The Exercise Benefits/Barriers Scale (EBBS), developed by Sechrist et al. (1987), has been psychometrically validated in the general population and established the role of health beliefs in shaping physical activity behaviours. To address the specific clinical and psychosocial context of dialysis, the Dialysis Patient–Perceived Exercise Benefits and Barriers Scale (DPEBBS) was later developed (Zheng et al. 2010). Translated versions of the DPEBBS have also been validated and used in cross‐national studies, including studies in China (Zheng et al. 2010), the United Kingdom (Lightfoot et al. 2021) and Turkey (Taş and Akyol 2019). Existing evidence indicates that exercise‐related perceptions among patients undergoing HD are shaped by cultural norms, healthcare practices and societal beliefs surrounding chronic illness, ageing and physical activity. For example, Lightfoot et al. (2021) examined how patients undergoing HD in the United Kingdom conceptualise exercise within the context of chronic illness, highlighting culturally embedded beliefs regarding appropriateness and safety. These findings highlight the importance of examining exercise‐related perceptions within specific cultural and healthcare contexts. Accordingly, the development of a Japanese version of the DPEBBS (JDPEBBS) is warranted to ensure culturally meaningful assessment and support future cross‐national research.

Beyond quantitative validation studies, qualitative research provides insights into how individuals receiving HD perceive exercise. Jhamb et al. (2016) reported that fatigue and dialysis‐related time limitations coexist with beliefs about ageing, the need for rest and perceptions of bodily vulnerability. Other studies have similarly shown that exercise perceptions are shaped by physical symptoms as well as broader psychosocial and cultural beliefs (Heiwe and Tollin 2012; Ghafourifard et al. 2021). Together, these findings suggest that exercise‐related perceptions reflect a complex interplay of physical, psychological and sociocultural factors.

Within the HPM framework, perceived benefits and barriers are inherently context‐dependent and vary substantially across cultural settings. Applying exercise perception scales developed in other countries without rigorous cultural adaptation risks failing to capture context‐specific cognitive and psychosocial determinants of physical activity among people receiving HD in Japan. Despite growing international evidence supporting the DPEBBS, no culturally validated Japanese version currently exists. This gap constrains both research and clinical practice.

Accordingly, this study aimed to develop a JDPEBBS and evaluate its psychometric properties through translation, cultural adaptation and validation in a representative sample of people receiving HD in Japan. Establishing such a tool is essential for identifying modifiable psychological factors, informing behavioural interventions and integrating patient‐reported perceptions into person‐centred HD care.

## 3 Materials and Methods

### 3.1 Study Design

This methodological study comprised two phases: (1) translation and cultural adaptation of the DPEBBS into Japanese and (2) psychometric evaluation of the JDPEBBS using a self‐administered survey among people receiving HD. The translation process followed internationally recognised guidelines, and the psychometric evaluation assessed its validity and reliability.

### 3.2 Translation and Cultural Adaptation

The JDPEBBS was developed in accordance with the ISPOR Task Force's ‘Principles of Good Practice for the Translation and Cultural Adaptation Process for Patient‐Reported Outcomes (PRO) Measures’ (Wild et al. 2005). The procedure included preparation, forward translation, reconciliation, back translation, harmonisation, cognitive debriefing and finalisation.

#### 3.2.1 Preparation and Forward Translation

Permission to translate the original instrument was obtained from Professor Li‐Ming You, who reviewed the key stages of the adaptation process. Two bilingual professional translators, fluent in English and Japanese and experienced in translating PRO measures, independently produced the forward translations. These versions were reconciled into a single provisional translation through discussion among the translators and a panel of five researchers with clinical experience. The panel examined semantic equivalence, cultural appropriateness and conceptual consistency with the original scale.

#### 3.2.2 Back Translation and Harmonisation

A native English‐speaking translator blinded to the original DPEBBS performed a back translation to evaluate semantic and conceptual equivalence. The original developer reviewed the back‐translated version and confirmed conceptual fidelity. Harmonisation by the research team and developer ensured alignment with existing language versions, producing the first draft of the JDPEBBS.

#### 3.2.3 Cognitive Debriefing and Finalisation

Cognitive debriefing was conducted with eight individuals receiving HD, representing a range of ages, genders and socioeconomic backgrounds. Participants identified unclear items, leading to revisions to improve clarity. The term ‘arteriovenous fistula’ in item X21 was replaced with ‘shunt’, a term more commonly used in Japan. Proofreading ensured linguistic accuracy. The expert panel reviewed the entire translation process, including all forward and back translations and subsequent revisions, reaching consensus on content validity. The final JDPEBBS was used for psychometric testing.

### 3.3 Psychometric Evaluation

#### 3.3.1 Participants and Procedures

Participants were recruited through convenience sampling from dialysis facilities accredited by the Japanese Society for Dialysis Therapy. Of 49 facilities contacted, 12 participated. Eligible individuals were adults aged ≥ 18 years receiving HD for at least 1 year. Individuals with severe physical or mental health conditions or cognitive impairment affecting questionnaire completion were excluded.

Data were collected from February 2024 to November 2024. At each participating facility, staff explained the study purpose and distributed the self‐administered questionnaire to eligible participants during their outpatient visits. Participants completed the questionnaire independently at the medical facility or at home on a non‐dialysis day or prior to a dialysis session. Completed questionnaires were returned directly to the research team using a pre‐addressed return envelope.

A minimum of five participants per item with at least 100 participants was considered adequate (Mokkink et al. 2018). Questionnaires were distributed to 320 eligible patients across 12 facilities, assuming a 50% response rate and targeting a sample of 150 patients.

Participants completed the JDPEBBS, the Japanese version of the International Physical Activity Questionnaire (IPAQ), plus a demographic and clinical questionnaire. The JDPEBBS comprises 24 items: 12 assessing perceived benefits and 12 assessing perceived barriers to exercise. Items were rated on a four‐point Likert scale ranging from 1 (*strongly disagree*) to 4 (*strongly agree*). Barrier items were reverse‐coded. Total scores range from 24 to 96, with higher scores indicating greater perceived benefits and fewer perceived barriers to exercise. The IPAQ assesses vigorous, moderate and walking physical activity over the previous week. Metabolic equivalent scores were calculated using the official scoring protocol (Craig et al. 2003; International Physical Activity Questionnaire Committee 2005). The IPAQ has been validated in Japanese populations (Tomioka et al. 2011; Kawabe et al. 2022).

#### 3.3.2 Statistical Analysis

All analyses were conducted using IBM SPSS Statistics version 27.0 J and IBM SPSS Amos version 29.0 J. Statistical significance was set at *p* < 0.05 (two‐tailed).

Structural validity was examined using exploratory and confirmatory factor analyses (EFA and CFA). EFA employed the principal factor method with ProMax rotation. Sampling adequacy was evaluated using the Kaiser–Meyer–Olkin (KMO) measure (> 0.50), and factorability was confirmed using Bartlett's test of sphericity (*p* < 0.05). CFA evaluated model fit using the comparative fit index (CFI), Tucker–Lewis index (TLI), root mean square error of approximation (RMSEA) and standardised root mean square residual (SRMR). Acceptable thresholds were defined as CFI ≥ 0.90, TLI ≥ 0.90 (Hair et al. 2009), RMSEA ≤ 0.06 and SRMR ≤ 0.08 (de Vet et al. 2011).

Concurrent validity was evaluated using Spearman's rank correlation coefficients between JDPEBBS and IPAQ total scores. Correlation coefficients ≥ 0.30 with *p* < 0.01 were considered indicative of adequate concurrent validity (Hinkle et al. 2003).

Construct validity was examined by comparing JDPEBBS scores between participants with and without regular exercise habits using the Mann–Whitney *U* test.

Reliability was assessed using internal consistency and item‐level performance. Cronbach's alpha coefficients ≥ 0.70 indicated acceptable internal consistency (de Vet et al. 2011). Item–total correlations were computed as the correlation between each item and the total score excluding that item, with values ≥ 0.30 indicating adequate item discrimination (de Vet et al. 2011).

### 3.4 Ethical Considerations

Ethical approval was obtained from the relevant institutional review board (Approval No. 2023‐012‐711099). Facility directors granted additional permission. Written informed consent was obtained from all participants, who were informed of their right to withdraw at any time prior to data collection.

## 4 Results

A total of 320 questionnaires were distributed to eligible participants, of which 156 were returned (response rate: 48.8%). After excluding six incomplete questionnaires, responses from 150 individuals receiving HD were retained for analysis (valid response rate: 96.2%). A valid response was defined as a questionnaire with complete JDPEBBS item data.

### 4.1 Participant Characteristics

The demographic and clinical characteristics of the 150 participants are summarised in Table 1. The mean age was 68.3 years (SD = 11.6), and most were male (*n* = 106, 71.1%). The average duration of HD treatment was 10.3 years (SD = 9.9). The primary causes of end‐stage kidney disease were chronic glomerulonephritis (*n* = 52; 34.7%), diabetic nephropathy (*n* = 44; 29.3%) and polycystic kidney disease (*n* = 14; 9.3%). Fifty‐six participants (37.3%) reported regular exercise.

**Table 1:** Participants' demographic and clinical characteristics.

Variable	*N*	*n*	%	Mean ± SD
Age (years)	150			68.26 ± 11.56
Gender	149			
Male		106	71.1	
Female		43	28.9	
Education level	149			
Primary school or junior middle school		21	14.1	
Senior middle school		99	66.4	
College/university		29	19.5	
Marital status	148			
Married		111	75.0	
Not married		37	25.0	
Employment status	149			
Employed full time		41	27.5	
Employed part time		17	11.4	
Not employed		91	61.1	
Exercise	150			
Yes		56	37.3	
No		94	62.7	
Primary diagnosis	150			
Diabetic nephropathy		44	29.3	
Chronic glomerulonephritis		52	34.7	
Nephrosclerosis		11	7.3	
Polycystic kidney disease		14	9.3	
Pyelonephritis		6	4.0	
Interstitial nephritis		2	1.3	
Autoimmune		5	3.3	
Other		18	12.0	
Dialysis duration (years)	148			10.25 ± 9.94

### 4.2 Validity

#### 4.2.1 Structural Validity

EFA indicated sampling adequacy (KMO = 0.876) and a significant Bartlett's test of sphericity (*p* < 0.01). Although the original six‐factor structure was not fully replicated, and several items were loaded on different factors, detailed EFA results are provided in the supplementary materials. As shown in Table S1, most items demonstrated factor loadings consistent with the original DPEBBS; however, several items (X7, X15, X17 and X12) loaded more strongly on factors different from those proposed in the original scale. These items are presented in bold outside the original factor groupings in the supplementary table, reflecting discrepancies between the original factor allocation and the present EFA findings.

**Figure 1:**
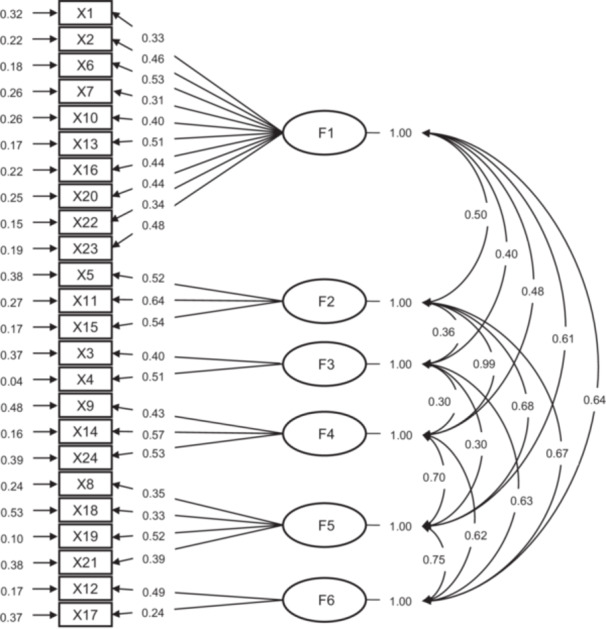
Factor structure of the Japanese version of the Dialysis Patient‐Perceived Exercise Benefits and Barriers Scale. This figure illustrates the six‐factor structure of the JDPEBBS identified through factor analysis. Items (X1–X24) are grouped according to their respective latent factors (F1–F6) with standardised factor loadings displayed for each item.

CFA was conducted based on the six‐factor structure reported in the original validation study. The first‐order model demonstrated acceptable fit (CFI = 0.922, TLI = 0.910, RMSEA = 0.055 and SRMR = 0.062), supporting the structural validity of the JDPEBBS (Figure 1).

The second‐order model, in which the six first‐order factors loaded onto one higher order factor, showed poorer fit (CFI = 0.889, TLI = 0.876, RMSEA = 0.065 and SRMR = 0.077) and was not retained.

#### 4.2.2 Concurrent Validity

Spearman's rank correlation analysis showed a significant association between the JDPEBBS and IPAQ total scores (*r* = 0.379, *p* < 0.01), exceeding the threshold of *r* ≥ 0.30 (Table 2).

**Table 2:** Correlations of the JDPEBBS and IPAQ (*N* = 150).

JDPEBBS variables	IPAQ			
	TPA	WPA	MPA	VPA
Total JDPEBBS	0.379**	0.315**	0.313**	0.142
F1	0.305**	0.210*	0.319**	0.204*
F2	0.342**	0.324**	0.173*	0.108
F3	0.153	0.111	0.033	0.02
F4	0.332**	0.280**	0.270**	0.079
F5	0.266**	0.241**	0.164*	0.089
F6	0.164*	0.175*	0.131	−0.125

Abbreviations: IPAQ, International Physical Activity Questionnaire; JDPEBBS, Japanese version of the Dialysis Patient‐Perceived Exercise Benefits and Barriers Scale; MPA, moderate intensity physical activity; TPA, total physical activity; VPA, vigorous‐intensity physical activity; WPA, walking physical activity.

* *p* < 0.05

** *p* < 0.01.

#### 4.2.3 Construct Validity (Known‐Groups Validity)

Known‐groups validity was examined by comparing participants who reported regular exercise (*n* = 56) with those who did not (*n* = 94). The exercise group had a significantly higher total JDPEBBS score (mean = 74.21, SD = 7.50) than the non‐exercise group (mean = 66.28, SD = 8.73; *p* < 0.001). Significant between‐group differences were observed across all six subscales (Table 3).

**Table 3:** Construct validity as comparison of mean JDPEBBS score between subgroups of exercise (*N* = 150).

	Exercise group (*n* = 56)		No exercise group (*n* = 94)		
	Mean	Median	Mean	Median	*p*‐Value
	SD	IQR	SD	IQR	Mann–Whitney *U* test
Total scale	74.21	73	66.28	66	< 0.001
	7.50	12	8.73	10	
Factor 1	30.61	30	26.89	28	< 0.001
	3.85	6	4.31	4	
Factor 2	9.14	9	7.80	8	< 0.001
	1.45	2	2.07	3	
Factor 3	6.55	6	6.15	6	0.023
	1.06	1	1.13	1	
Factor 4	9.57	9	8.57	9	0.001
	1.51	3	1.97	3	
Factor 5	12.63	12	11.62	12	0.001
	1.76	3	1.97	1	
Factor 6	5.71	6	5.24	5	0.006
	0.97	1	1.04	1	

Abbreviations: IQR, interquartile range; SD, standard deviation.

### 4.3 Reliability

The JDPEBBS demonstrated excellent internal consistency, with an overall Cronbach's alpha of 0.901. All corrected item–total correlations exceeded 0.30, indicating adequate item‐level discrimination (Table 4).

**Table 4:** Item means, standard deviations, item–total correlations and Cronbach's *α* coefficient if item deleted (*N* = 150).

Item		Mean	SD	Corrected item–total correlation	Cronbach's *α* coefficient if item deleted
X1	Exercise helps reduce my total medical costs.	2.81	0.652	0.400	0.900
X2	Exercise helps reduce my body pain.	2.79	0.661	0.514	0.897
X3	Exercise postpones a decline in body function.	3.12	0.732	0.314	0.902
X4	Exercise prevents muscular atrophy.	3.18	0.544	0.422	0.899
X5	Frequent tiredness impedes my exercise participation.	2.55	0.808	0.491	0.898
X6	Exercise improves my mood.	2.91	0.675	0.652	0.894
X7	Exercise improves bone diseases.	2.68	0.594	0.474	0.898
X8	Exercise is adverse to health of dialysis patients.	3.26	0.607	0.428	0.899
X9	I worry about a fall during exercise.	2.68	0.822	0.413	0.900
X10	Exercise improves my appetite.	2.87	0.652	0.464	0.898
X11	Frequent lower extremity muscle fatigue impedes my exercise participation.	2.69	0.827	0.598	0.895
X12	I lack an understanding of the benefits of exercise.	3.11	0.640	0.582	0.896
X13	Exercise helps me lead an optimistic and active life.	2.97	0.655	0.650	0.894
X14	Exercise is not suitable for me since I have other comorbidities.	3.06	0.697	0.633	0.895
X15	Body pain impedes my exercise participation.	3.06	0.688	0.633	0.895
X16	Exercise improves my quality of life.	3.03	0.645	0.531	0.897
X17	I lack an understanding of the knowledge on how to carry out exercise.	2.31	0.655	0.366	0.900
X18	I worry that exercise may make me feel thirsty.	2.78	0.802	0.300	0.903
X19	Exercise is not suitable for me since I have kidney disease.	3.07	0.609	0.660	0.894
X20	Exercise can keep my body weight at steady level.	2.55	0.671	0.551	0.896
X21	I worry that exercise may affect my arteriovenous fistula.	2.89	0.728	0.426	0.899
X22	Exercise helps enhance my self‐care abilities.	2.91	0.517	0.573	0.897
X23	Exercise will keep me from having other diseases (e.g., cold).	2.77	0.649	0.606	0.895
X24	Outdoor exercise adds burden to my family since I need their company while I am out.	3.21	0.822	0.453	0.899

## 5 Discussion

### 5.1 Principal Findings

This study is the first to translate, culturally adapt and validate the DPEBBS for use in Japan. The resulting instrument, the JDPEBBS, retained the original six‐factor structure and demonstrated robust psychometric properties. Specifically, the JDPEBBS demonstrated moderate concurrent validity with physical activity levels, sound known‐groups validity and high internal consistency. These findings are consistent with prior validations of the DPEBBS conducted in other cultural contexts, supporting the tool's cross‐cultural applicability among patients undergoing HD.

### 5.2 Validity

Structural validity of the JDPEBBS was examined using both EFA and CFA. Although the EFA did not fully reproduce the original six‐factor structure of the DPEBBS, the CFA showed acceptable fit for the Japanese data (CFI = 0.922, TLI = 0.910, RMSEA = 0.055 and SRMR = 0.062), supporting the structural validity of the original model in this context. These CFA results are comparable to those reported in previous validation studies (Zheng et al. 2010; Taş and Akyol 2019). The cross‐cultural applicability of the DPEBBS is further supported by studies conducted in China (Hu et al. 2025; Huang et al. 2023) and Saudi Arabia (Almaimani et al. 2026), which reported associations between exercise perceptions and physical activity participation among HD patients.

By contrast, the EFA revealed deviations from the original factor configuration, particularly for items X7, X15, X17 and X12. Such discrepancies between exploratory and confirmatory analyses are commonly observed in cross‐cultural adaptation studies and generally interpreted as reflecting cultural or clinical differences in cognitive organisation rather than deficiencies in translation or measurement quality (Beaton et al. 2000; Wild et al. 2005). In this study, item X7 also deviated from the original structure, exhibiting cross‐loadings across benefit‐ and knowledge‐related factors. This pattern suggests that the bone‐related benefits of exercise may be perceived not as an intuitive general benefit but as a medically contingent outcome requiring adequate understanding and reassurance, particularly given the heightened concerns about bone fragility and treatment safety among people receiving HD in Japan. Item X15 clustered with concerns related to falling, comorbidities and perceived burden on family members, suggesting that pain may be perceived as a marker of risk and social burden rather than as an isolated physical symptom. Item X17 loaded together with tiredness and muscle fatigue, indicating that a lack of knowledge about exercise may reflect reduced self‐efficacy associated with physical limitations. Item X12 clustered with kidney disease–specific concerns, such as thirst and disease‐related symptoms, suggesting that perceptions of exercise benefits are evaluated primarily through a disease‐centred lens focused on symptom management and treatment safety. These culturally specific patterns are consistent with findings from China, where lack of exercise knowledge, muscle fatigue and tiredness were identified as predominant barriers (Huang et al. 2023; Hu et al. 2025), and from the United Kingdom, where tiredness and comorbidities were the most frequently reported barriers (Lightfoot et al. 2021). Collectively, these findings suggest that physical symptoms and disease‐specific concerns consistently shape exercise perceptions across HD populations, albeit with cultural nuance in their relative salience.

Despite the culturally specific clustering patterns in EFA, CFA findings indicated that the original six‐factor structure remained statistically acceptable and clinically interpretable in the Japanese context. As the primary aim of this study was cultural adaptation rather than scale redevelopment, and to maintain international comparability with existing versions of DPEBBS, the original factor structure was retained for the JDPEBBS.

Construct validity was further supported by known‐groups validation, with participants engaging in regular exercise demonstrating significantly higher JDPEBBS total and subscale scores than those who did not. Concurrent validity was also confirmed by a significant association between JDPEBBS scores and physical activity levels measured using the IPAQ (*r* = 0.379, *p* < 0.01). This exceeded the predefined criterion of *r* ≥ 0.30, commonly considered indicative of adequate construct validity in behavioural and health‐related research (Hinkle et al. 2003; de Vet et al. 2011). These findings align with theoretical frameworks emphasising perceptual and cognitive influences on exercise behaviour (Bandura 1991; Deci and Ryan 2008).

### 5.3 Reliability

Reliability of the JDPEBBS was assessed using internal consistency and item‐level performance. The total scale demonstrated excellent internal consistency, with a Cronbach's alpha of 0.901, exceeding the predefined criterion of 0.70 (de Vet et al. 2011). This finding indicates reliable measurement of exercise‐related benefits and barriers.

All corrected item–total correlations exceeded 0.30 (de Vet et al. 2011), indicating adequate item discrimination and confirming that all items contributed meaningfully to the total score. No item showed weak associations with the overall scale, supporting appropriate item functioning.

The internal consistency observed in this study is comparable to that reported in the original validation study conducted by Zheng et al. (2010) and in subsequent cross‐cultural validations. Recent studies using the DPEBBS have reported similar levels of internal consistency, with Cronbach's alpha values of 0.87 in a large multicentre Chinese study (Hu et al. 2025) and 0.92 for the benefits subscale and 0.86 for the barriers subscale in a study conducted in Saudi Arabia (Almaimani et al. 2026), supporting the cross‐cultural reliability of the scale.

Reliability was assessed solely in terms of internal consistency and item performance. Although these indices provide important information regarding the degree to which items are interrelated at a single point in time, they do not address the temporal stability of the instrument. Therefore, future studies should examine test–retest reliability to determine whether the JDPEBBS scores remain stable over time, particularly when the scale is intended for use in longitudinal research or intervention studies involving people receiving HD.

### 5.4 Cultural and Clinical Relevance

Cross‐cultural validity extends beyond statistical model fit and requires that an instrument's underlying construct be conceptually meaningful for the target population (Beaton et al. 2000). Exercise‐related perceptions are shaped by sociocultural and clinical norms surrounding healthcare interactions, chronic illness and ageing, influencing how patients perceive fatigue and physical exertion. Qualitative research in HD populations shows that fatigue and physical vulnerability are often viewed as unavoidable consequences of chronic illness or ageing, leading patients to perceive exercise as inappropriate or potentially harmful rather than as a modifiable behaviour (Jhamb et al. 2016). This perception appears consistent across cultures: tiredness and muscle fatigue were among the most frequently reported barriers in studies conducted in the United Kingdom (Lightfoot et al. 2021), China (Huang et al. 2023) and Saudi Arabia (Almaimani et al. 2026), underscoring the universality of symptom‐driven barriers across HD populations.

Several adaptations introduced during the translation process enhanced comprehensibility while preserving clinical accuracy. For instance, replacing the term ‘arteriovenous fistula’ with ‘shunt’ reflected terminology more commonly used and understood by people receiving HD in Japan. In addition, feedback from cognitive debriefing informed minor wording revisions to align items with Japanese idiomatic expressions and health‐related beliefs. These procedures are consistent with best practices in cross‐cultural instrument development (Wild et al. 2005).

Consistent with prior work on attitude scales for dietary therapy among people receiving HD (Onbe and Kanda 2018), successful adaptation of patient‐reported outcome measures depends not only on linguistic accuracy but also on conceptual alignment with individuals' lived experiences. In this respect, the JDPEBBS provides a culturally attuned instrument capable of capturing nuanced psychosocial factors that influence exercise behaviour among people receiving HD in Japan. Given that physical inactivity in this population is associated with cardiovascular morbidity, reduced functional capacity and impaired quality of life, a psychometrically sound and culturally appropriate tool for identifying modifiable exercise‐related perceptions is of considerable clinical importance.

### 5.5 Limitations and Future Research

This study has several limitations. Participants were recruited through convenience sampling from facilities that voluntarily agreed to participate, potentially introducing selection bias. Physical activity was assessed using self‐report measures and may be subject to recall and social desirability bias. In addition, test–retest reliability and responsiveness to change were not examined.

Future research should explore how culturally embedded values shape exercise perceptions among people receiving HD in Japan and assess predictive validity by evaluating associations between JDPEBBS scores and subsequent exercise adherence, functional outcomes or hospitalisation risk.

## 6 Implications for Clinical Practice

The JDPEBBS has practical implications for dialysis nurses, physiotherapists and interdisciplinary care teams. The scale helps identify patients who perceive high barriers to exercise or low anticipated benefits, enabling targeted education, counselling or referrals to appropriate physical activity programmes. It may also be integrated into routine clinical pathways, such as annual health assessments or pre‐rehabilitation screening.

Given the frequency of patient–nurse interactions in HD care, the JDPEBBS may support motivational interviewing and tailored behavioural counselling in HD care. When fear of fatigue or injury is identified as a primary barrier, clinicians can collaboratively develop a low‐intensity or supervised exercise plan appropriate to patients' physical capacity and confidence.

At the organisational level, aggregated JDPEBBS data can inform quality improvement initiatives in dialysis facilities. Monitoring exercise‐related perceptions may help evaluate exercise promotion strategies and rehabilitative resource allocation.

## 7 Conclusion

This study developed and validated the JDPEBBS and demonstrated satisfactory psychometric properties among Japanese patients receiving HD. The scale showed acceptable structural validity, adequate construct validity and excellent internal consistency, supporting its reliability and applicability in the Japanese context.

By providing a standardised and culturally adapted measure of exercise‐related perceptions, the JDPEBBS addresses an important gap in renal care research and practice in Japan. These findings underscore the relevance of cognitive and perceptual factors, as conceptualised within the HPM, for shaping exercise behaviour among individuals receiving HD.

The JDPEBBS may support nurses and interdisciplinary renal care teams in identifying modifiable psychological barriers to exercise and informing individualised counselling and rehabilitation strategies. Future studies should examine temporal stability, responsiveness and predictive validity.

## Author Contributions

**Ryota Kumakura:** conceptualisation, methodology, validation, formal analysis, investigation, data curation, writing – original draft, writing – review and editing, project administration. **Keiko Tasaki:** conceptualisation, methodology, validation, formal analysis, writing – review and editing, supervision, project administration. **Tomomi Horiguchi:** conceptualisation, methodology, validation, formal analysis, writing – review and editing, supervision, project administration. **Yuya Asada:** conceptualisation, methodology, validation, writing – review and editing. **Yukari Fujita:** conceptualisation, methodology, validation, writing – review and editing. **Noboru Fujino:** conceptualisation, methodology, validation, writing – review and editing.

## Ethics Statement

Ethical approval was obtained from the Institutional Review Board of Kanazawa University (Approval No. 2023‐012‐711099).

## Conflicts of Interest

The authors declare no conflicts of interest.

## Supporting information


Supporting File

## Data Availability

Data supporting the findings of this study are available from the corresponding author upon request. The data are not publicly available due to restrictions requested by the participating hospitals.
